# Observations on Meningococcus Carriers and on the Bacteriology of Epidemic Meningitis

**Published:** 1918-11

**Authors:** 


***Observations on Meningococcus Carriers and on the Bacteriology of Epidemic Meningitis.*** By George Mathers and Russell D. Herrold. *Journal of Infectious Diseases,* June, 1918.

It seems probable that the number of susceptible individuals in any community is very small, and that crowded living quarters, fatigue, exposure, and frequent changes in the population are factors which favor the development of the infection. The most important procedure relied on for the control of epidemic meningitis involved the identification and detention of healthy meningococcus carriers.

As soon as a case of meningitis occurred, the immediate contacts were examined bacteriologically, and the carriers identified and isolated. If more than one case occurred in an organization, the entire organization was examined for carriers. It was found that 3-6 o/o of the men examined harbored meningococci in the secretions of the nose and throat. The number of carriers in different groups of men varied.

The majority of these carriers, however, were of the temporary type; only 1-2 o/o of the total number of suspects examined proved to be chronic carriers. Chronic meningococcus carriers, as distinguished from the temporary type, often harbored great numbers of meningococci in the secretions of the nose and throat. The number of carriers was found to be high among those coming in contact with meningitis cases.

In a study of the biologic reactions of 150 strains of meningococci, from different sources, two large biologic groups were differentiated by means of macroscopic agglutination tests, using monovalent serums. The agglutination reactions were in most instances definite and specific, but a number of atypical and inagglutinable strains were met with in each group. The atypical strains, however, did not differ enough from the other members of the group to warrant different classifications as determined by agglutination. The classification of the inagglutinable strains was accomplished by means of agglutination with monovalent serums prepared from these strains; these serums yielded specific reactions, with organisms of one or the other main types.

It seems important to emphasize the distinction between the two types of meningococcus carriers. The chronic carriers, individuals yielding positive nasopharyngeal cultures over long periods of time, were few in number, and only in rare instances developed meningitis. In the cultures from these persistent carriers, the meningococci in most cases were present in predominating numbers. Indeed, many pure cultures were obtained in such cases. In our experience the connection between chronic meningococcus carriers in cases of meningitis has been striking in many instances, and has seemed to us to indicate the necessity of special attention being given to this type of carrier in all epidemics of this disease.

